# Blocking CCN2 Reduces Established Bone Loss Induced by Prolonged Intense Loading by Increasing Osteoblast Activity in Rats

**DOI:** 10.1002/jbm4.10783

**Published:** 2023-06-16

**Authors:** Alex G Lambi, Michele Y Harris, Mamta Amin, Patrice G Joiner, Brendan A Hilliard, Soroush Assari, Steven N Popoff, Mary F Barbe

**Affiliations:** ^1^ Department of Orthopedics and Rehabilitation University of New Mexico Albuquerque NM USA; ^2^ Center for Translational Medicine, Lewis Katz School of Medicine Temple University Philadelphia PA USA; ^3^ Exponent, Inc. Philadelphia PA USA; ^4^ Department of Biomedical Education and Data Science, Lewis Katz School of Medicine Temple University Philadelphia PA USA

**Keywords:** CCN2, CTGF, MUSCULOSKELETAL DISORDERS, OSTEOPENIA, OVERUSE, PAMREVLUMAB

## Abstract

We have an operant model of reaching and grasping in which detrimental bone remodeling is observed rather than beneficial adaptation when rats perform a high‐repetition, high‐force (HRHF) task long term. Here, adult female Sprague–Dawley rats performed an intense HRHF task for 18 weeks, which we have shown induces radial trabecular bone osteopenia. One cohort was euthanized at this point (to assay the bone changes post task; HRHF‐Untreated). Two other cohorts were placed on 6 weeks of rest while being simultaneously treated with either an anti‐CCN2 (FG‐3019, 40 mg/kg body weight, ip; twice per week; HRHF‐Rest/anti‐CCN2), or a control IgG (HRHF‐Rest/IgG), with the purpose of determining which might improve the trabecular bone decline. Results were compared with food‐restricted control rats (FRC). MicroCT analysis of distal metaphysis of radii showed decreased trabecular bone volume fraction (BV/TV) and thickness in HRHF‐Untreated rats compared with FRCs; responses improved with HRHF‐Rest/anti‐CCN2. Rest/IgG also improved trabecular thickness but not BV/TV. Histomorphometry showed that rest with either treatment improved osteoid volume and task‐induced increases in osteoclasts. Only the HRHF‐Rest/anti‐CCN2 treatment improved osteoblast numbers, osteoid width, mineralization, and bone formation rate compared with HRHF‐Untreated rats (as well as the latter three attributes compared with HRHF‐Rest/IgG rats). Serum ELISA results were in support, showing increased osteocalcin and decreased CTX‐1 in HRHF‐Rest/anti‐CCN2 rats compared with both HRHF‐Untreated and HRHF‐Rest/IgG rats. These results are highly encouraging for use of anti‐CCN2 for therapeutic treatment of bone loss, such as that induced by chronic overuse. © 2023 The Authors. *JBMR Plus* published by Wiley Periodicals LLC on behalf of American Society for Bone and Mineral Research.

## Graphical abstract

Adult female rats performed an intense high repetition high force task for 18 weeks. One cohort was euthanized at 18 weeks (HRHF Untreated), two other cohorts rested for 6 weeks while being treated with an anti‐CCN2 or an IgG. Results were compared to controls. MicroCT analysis of distal metaphysis of radii demonstrated decreased trabecular bone volume fraction and thickness in HRHF‐Untreated rats; responses improved with HRHF‐Rest/anti‐CCN2. Rest/IgG also improved trabecular thickness, but not volume. Serum ELISA showed increased osteocalcin and decreased CTX‐1 in HRHF‐Rest/anti‐CCN2 rats, compared to both HRHF‐Untreated and HRHF‐Rest/IgG rats.

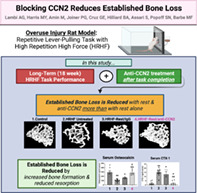

## Introduction

CCN2 (cellular communication network factor 2, also known as connective tissue growth factor [CTGF]) is a matricellular protein that is required for normal skeletogenesis, including regulation of osteoblast and osteoclast differentiation and function.^[^ ^1^, ^2^, ^3^, ^4^ ^]^ A number of cells in the bone microenvironment secrete this protein, including osteocytes, osteoblasts, chondrocytes, and endothelial cells.^[^ ^5^, ^6^, ^7^ ^]^ Like other CCN proteins, CCN2 has a multimodular structure including an insulin‐like growth factor (IGF)‐binding domain, von Willebrand type C (vWC) domain, thrombospondin (TSP)‐1 domain, and a C‐terminal domain containing a putative cysteine knot. The mosaic structure of the protein allows it to play complex, regulatory biological roles in bone.^[^ ^8^ ^]^ These include CCN2 directly binding to cell‐surface receptors (eg, integrins) and mediating cell‐surface binding of cytokines (eg, vascular endothelial growth factors) to stimulate signal transduction.^[^ ^9^, ^10^ ^]^ CCN2 also binds components of the extracellular matrix (eg, proteoglycans) that can assist with cell adhesion and motility as well as matrix turnover.^[^ ^11^, ^12^ ^]^ Through these interactions, CCN2 serves a critical role in osteogenesis, chondrogenesis, and angiogenesis necessary for skeletal development and homeostasis.

A clear role for CCN2 in proper skeletal formation has been identified in studies using global CCN2 knockout (KO) mice.^[^ ^3^, ^4^ ^]^ CCN2 KO mice show site‐specific malformations in craniofacial, vertebral, and long bones. These changes are associated with alterations in matrix production and formation of cartilage and bone. An excess of CCN2 in the bone microenvironment is also detrimental as its constitutive overexpression in osteoblasts results in their decreased differentiation,^[^ ^13^ ^]^ as well as osteopenia in a transgenic mouse model.^[^ ^14^, ^15^ ^]^ One potential mechanism is a negative regulatory role with bone morphogenetic protein (BMP)‐2.^[^ ^13^ ^]^ In addition to its role in osteoblasts, CCN2 is also necessary for osteoclast formation and function. CCN2 is necessary for receptor activator of NF‐κB ligand (RANK‐L)‐induced osteoclastogenesis^[^ ^16^, ^17^ ^]^ and enhances RANK‐RANK‐L signaling. CCN2 also binds to osteoprotegerin (OPG), preventing the inhibitory effect of OPG on osteoclast formation. Exogenous administration of CCN2 in an osteoarthritic cell model results in osteoclast formation.^[^ ^18^ ^]^ These studies show that CCN2 functions in bone formation as well as bone resorption in a cell‐ and context‐specific fashion.

We have a model of upper‐extremity overuse injuries, in which rats perform a reaching and lever‐bar pulling task. The rats, motivated by obtaining a food reward, learn to pull the lever at defined reach rates and target forces.^[^ ^19^ ^]^ We have shown exposure‐dependent changes in bone, with anabolic responses found in the distal radius when young adult rats perform a moderate high‐repetition, low‐force task for 12 to 18 weeks as a result of increased osteoblast numbers and bone formation.^[^ ^19^, ^20^ ^]^ In contrast, when young adult rats perform an intense high‐repetition, high‐force task (HRHF; 4 reaches/min at ~50% of their maximum grasping force) for 3, 12, or 18 weeks, a catabolic response is observed in the distal radius as a result of increased osteoclast numbers and activity.^[^ ^19^, ^20^, ^21^, ^22^ ^]^

As CCN2 has been shown to play an important role in normal bone metabolism, we have previously assessed CCN2 in the distal radial trabecular bone after 3 weeks of HRHF task performance. Although both control and HRHF rats showed CCN2 expression in cells lining the bone and in marrow spaces, HRHF rats also showed increased deposition within the bone matrix.^[^ ^7^ ^]^ A monoclonal anti‐CCN2 antibody, known as FG‐3019 or Pamrevlumab, is being developed for use in treating multiple diseases, including muscular dystrophy and idiopathic pulmonary fibrosis.^[^ ^23^, ^24^, ^25^ ^]^ We obtained this antibody and applied it to our model. When the anti‐CCN2 antibody was administered during short‐term HRHF task performance, 3‐week anti‐CCN2‐treated task rats showed higher trabecular bone volume fraction as a result of reduced osteoclast numbers compared with untreated task rats. Yet, similar increases in osteoblast numbers and osteoid volume were observed in response to the loading in both task groups compared with resting control animals. These data suggest that blocking CCN2 inhibits osteoclastogenesis and activity after short‐term intense loading, without affecting osteoblast function.^[^ ^7^ ^]^ We have yet to assess whether blocking CCN2 signaling could recover established trabecular bone loss that occurs after long‐term reaching and grasping.

Thus, we sought here to examine for the first time, in our laboratory or in the literature, whether blocking CCN2 signaling using a targeted monoclonal antibody would enhance the recovery of forelimb bone after long‐term (18‐week) HRHF task performance that we have shown leads to a significant loss in trabecular bone.^[^ ^20^ ^]^ We examined the effect of treatment with the anti‐CCN2 monoclonal antibody compared with treatment with a human IgG control, each administered during a 6‐week rest recovery period. Based on our prior findings of improvements in trabecular bone after anti‐CCN2 treatment during short‐term HRHF task performance,^[^ ^7^ ^]^ we hypothesized that Rest/anti‐CCN2 would result in a reduction of established bone loss by reducing osteoclastogenesis and activity more than Rest/IgG treatment.

## Materials and Methods

### Overview of animals

All experiments were approved by the Temple University Institutional Animal Care and Use Committee (Temple University IACUC, animal care and use protocol # 4787) in compliance with NIH guidelines for the humane care and use of laboratory animals. Studies were conducted on 32 young adult, female, Sprague–Dawley rats (Taconic Bioscience, Germantown, NY, USA). Rats were 3 months of age at the beginning of the experiments and 7.5 to 9 months of age at their completion. Rat care details are as previously described.^[^ ^7^ ^]^

**Fig. 1:**
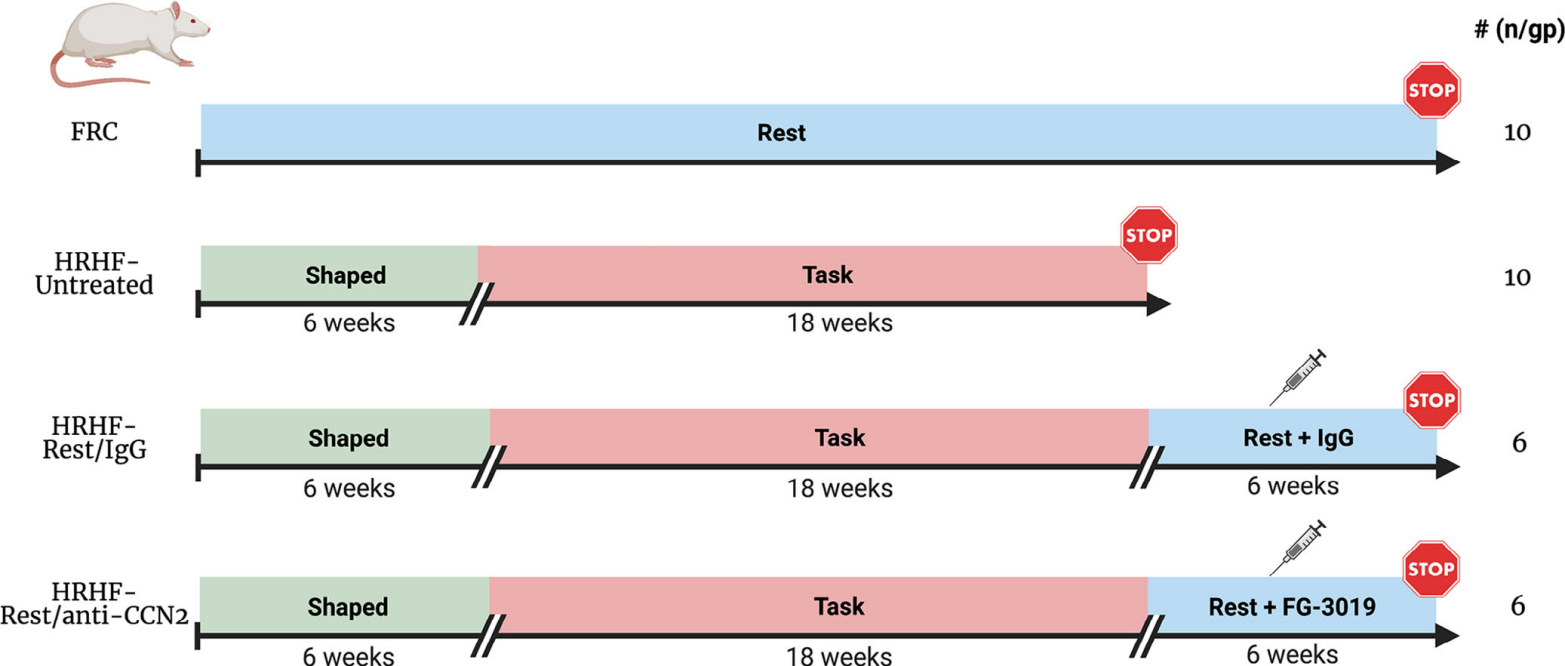
Experimental design. Controls included food‐restricted control rats (FRC) with no exposure to operant shaping or task performance. HRHF (high‐repetition, high‐force) task rats were similarly food restricted to motivate them to reach and grasp a lever bar for food pellet rewards. They were operantly shaped for 6 weeks to learn the high‐force lever‐bar pulling. They then went on to perform the HRHF task for 18 weeks. One subset of HRHF rats performed the task for 18 weeks before euthanasia and tissue collection (HRHF‐Untreated). Two subsets of 18‐week‐old HRHF rats were provided 6 weeks of rest after task cessation with either simultaneous systemic (ip) treatment with human IgG (HRHF‐Rest/IgG group) or anti‐CCN2 (HRHF‐Rest/anti‐CCN2 group).

Experimental design is shown in Figure 1. Controls included food‐restricted control rats (FRC) that were food restricted to 5% less than age‐matched normal control rats (the latter not included in this study), similar to task rats, yet no exposure to operant shaping or task performance. Twenty‐two rats were randomly chosen to undergo an initial operant shaping period for 6 weeks to learn a high‐force lever‐pulling task, before then going on to perform a HRHF lever‐pulling task for 18 weeks. Rats that had learned the task were randomly divided into three subcohorts. One subset of HRHF rats performed the task for 18 weeks before tissue collection (HRHF‐Untreated, *n* = 10). Two subsets of 18‐week‐old HRHF rats were provided 6 weeks of rest after task cessation with either simultaneous treatment with anti‐CCN2 (FG‐3019, 40 mg/kg body weight, i.p.; twice per week; HRHF‐Rest/anti‐CCN2 group, *n* = 6), or human IgG (HRHF‐Rest/IgG group, i.p., *n* = 6). One animal in the HRHF‐Rest/IgG group died from unknown reasons in task week 12 (this group was originally *n* = 6, thereby reducing the final number to *n* = 5 for this group). Results were compared with a control group of FRC rats (*n* = 10) receiving similar amounts of rat chow and food reward pellets as HRHF groups.

This is the third article from this series of animals. The previous studies focused on the effects of blocking CCN2 signaling in neural and musculotendinous tissues in this experimental model and design.^[^ ^26^, ^27^ ^]^

### Operant shaping for 6 weeks and behavioral task

Twenty‐two rats were randomly chosen to become HRHF task rats (this count includes the one dropout). These rats were operantly shaped across a 6‐week learning period in which they learned to perform a reaching and lever‐pulling task, as described.^[^ ^7^, ^19^ ^]^ Rats went on to perform the HRHF task, at 50% of their maximum pulling force, and a target of 4 reaches/min, for 2 h/d, in 30‐minute intervals with 1.5 hours rest breaks in between, 3 d/wk, for 18 weeks.^[^ ^20^, ^27^ ^]^ We have previously shown that the animals develop discomfort from the task and switch limbs or use both limbs simultaneously to pull on the lever bar in their attempt to garner a food reward pellet, beginning in task weeks 2 and 3.^[^ ^28^, ^29^, ^30^ ^]^ Thus, both right and left forelimbs were used.

HRHF task reach outcomes (mean number of reaches/min, mean grasp force in centi‐Newtons [cN] of force, mean grasp duration in milliseconds [ms], and mean reach impulse) were recorded continuously, as previously described.^[^ ^20^, ^31^ ^]^ Data are provided for task weeks 1, 3, 6, 9, 12, and 18. These data could not be generated for FRC rats as they did not perform the task, nor could these data be assessed in the rats resting after task cessation.

### Pharmacological treatments

Two subsets of 18‐week‐old HRHF task rats were provided 6 weeks of rest after task cessation, with simultaneous treatment of either: (i) human anti‐CCN2 monoclonal antibody (FG‐3019, a gift from FibroGen, Inc., San Francisco, CA, USA; 40 mg/kg body weight, i.p.; twice per week for 6 weeks); or (ii) human IgG, the vehicle for FG‐3019 (IgG, provided by FibroGen, Inc.; ip in matching volumes as used for the FG‐3019 drug; twice per week for 6 weeks), as described.^[^ ^15^, ^20^ ^]^ FG‐3019 has been previously characterized,^[^ ^32^, ^33^ ^]^ and effects in FRC rats reported.^[^ ^34^ ^]^

### Fluorochrome injections for bone growth measurement

In all rats in the study, calcein was injected at 9 days before euthanasia (10 mg/kg body weight, ip). Then, either calcein or alizarin red was injected at 2 days before euthanasia (10 mg/kg body weight, sc; most were injected with alizarin red as the second fluorochrome; two were inadvertently injected with calcein). The separately labeled fluorochromes assisted in analysis and photographic representation of dynamic histomorphometry.

### Tissue collection

Animals were deeply anesthetized with 5% isoflurane in oxygen and then euthanized by performing thoracotomy and cardiac puncture for blood collection using a 23G needle. Serum was harvested from blood, as described.^[^ ^27^ ^]^ Rats were then transcardially perfused with 4% paraformaldehyde, as described.^[^ ^27^ ^]^ A randomization schema was used so that a mix of right and left limbs was collected for each assay choice, with limbs used to reach collected from each HRHF group. For microCT and histomorphometry, forelimb bones were collected from one forelimb of 10 age‐matched FRC rats, and from limbs used to reach from 10 HRHF‐Untreated, 6 HRHF‐Rest/anti‐CCN2, and 5 HRHF‐Rest/IgG rats. For paraffin embedding and immunohistochemistry (to assay for CCN2 immunoexpression levels), forelimb bones were collected after fixation from 4 FRC and 4 HRHF‐Untreated rats (using limbs used to reach).

Thus, a total of 39 forelimb bones were used for this study: 14 from 10 FRC rats, 14 from 10 HRHF‐Untreated rats, 6 from 6 HRHF‐Rest/anti‐CCN2 rats, and 5 from 5 HRHF‐Rest/IgG rats.

### MicroCT imaging and analysis

After fixation, forelimb bones from 10 age‐matched FRC, 10 HRHF‐Untreated, 5 HRHF‐Rest/IgG, and 6 HRHF‐Rest/anti‐CCN2 rats were stored in phosphate‐buffered saline (PBS) with sodium azide until they underwent microCT scanning using previously described instrumentation and methods.^[^ ^20^, ^31^ ^]^ MicroCT scanning and analysis was performed, as previously described, in metaphyseal and diaphyseal regions of the radius,^[^ ^31^ ^]^ by an individual who was blinded to group assignment, according to published guidelines.^[^ ^35^ ^]^ The metaphyseal trabecular bone region of interest was delineated from 150 to 250 μm below the center of the distal growth plate. We focused on analyzing radial bone changes because the radius undergoes more task‐induced changes than the ulna in this task.^[^ ^20^, ^21^, ^22^ ^]^

### Staining and histomorphometry of bone

After microCT analysis, the same forelimb bones were processed for histomorphometry by embedding them in methyl methacrylate resin, sectioning them into 5‐μm longitudinal sections, before placing sections on charged slides (embedding and sectioning was performed by Bioquant Image Analysis Incorporation, Nashville, TN, USA). Unstained plasticized longitudinal sections with distal radial metaphyses were used to measure the previously injected calcein and alizarin red to determine dynamic histomorphometry parameters of trabecular bone microarchitecture of the distal radius, in accordance with the recommendations of the American Society for Bone and Mineral Research,^[^ ^36^ ^]^ for single‐labeled surfaces (sLS), double‐labeled surfaces (dLS), mineralizing surfaces (MS/BS), and bone formation rate (BFR/BS). This was performed in a region located 150 μm below the chondro‐osseous junction of the secondary spongiosa and 50 μm in from the surrounding cortical bone, using a Nikon E800 epifluorescent microscope (Nikon, Melville, NY, USA) with a customized X‐Y motorized stage (Applied Scientific Instrumentation, Eugene, OR, USA) and a digital camera (Gryphax Jenoptik, Jena, Germany), all interfaced with imaging software (Bioquant Osteo 2022, Bioquant Image Analysis) on a Windows 11 PC. Adjacent slides underwent Masson's trichrome or TRAP staining. Static histomorphometry parameters of trabecular bone microarchitecture were assayed in accordance with the recommendations of the American Society for Bone and Mineral Research,^[^ ^36^ ^]^ in the same trabecular region as described above, for osteoid volume (OV/BV), osteoid surface (OS/BS), number of osteoblasts per bone surface (N.Ob/BS), and osteoid width (O.Wi) in Masson's trichrome‐stained sections, and for osteoclast surface (Oc.S/BS) and number of osteoclasts per bone surface (N.Oc/BS) in TRAP‐stained slides. For all histomorphometry, the person carrying out the analyses was naive to group assignment.

### Immunohistochemistry for CCN2 and its quantification

Bones from 4 FRC and 4 HRHF‐Untreated rats were used to assay for CCN2 immunoexpression levels. For this, fixed bones were paraffin embedded after a 1‐month decalcification period (# NC9044643, StatLab Immunocal solution, Thermo Fisher Scientific, Waltham, MA, USA), sectioned, and then immunostained with an anti‐CCN2 antibody (CCN2/CTGF; #SC‐14939, Santa Cruz Biotechnology, Dallas, TX, USA), as well as Collagen type 1 (#C2456, Sigma, St. Louis, MO, USA) immunostaining and DAPI staining as counterstains, using previously described methods and validated antibodies.^[^ ^7^ ^]^

**Fig. 2:**
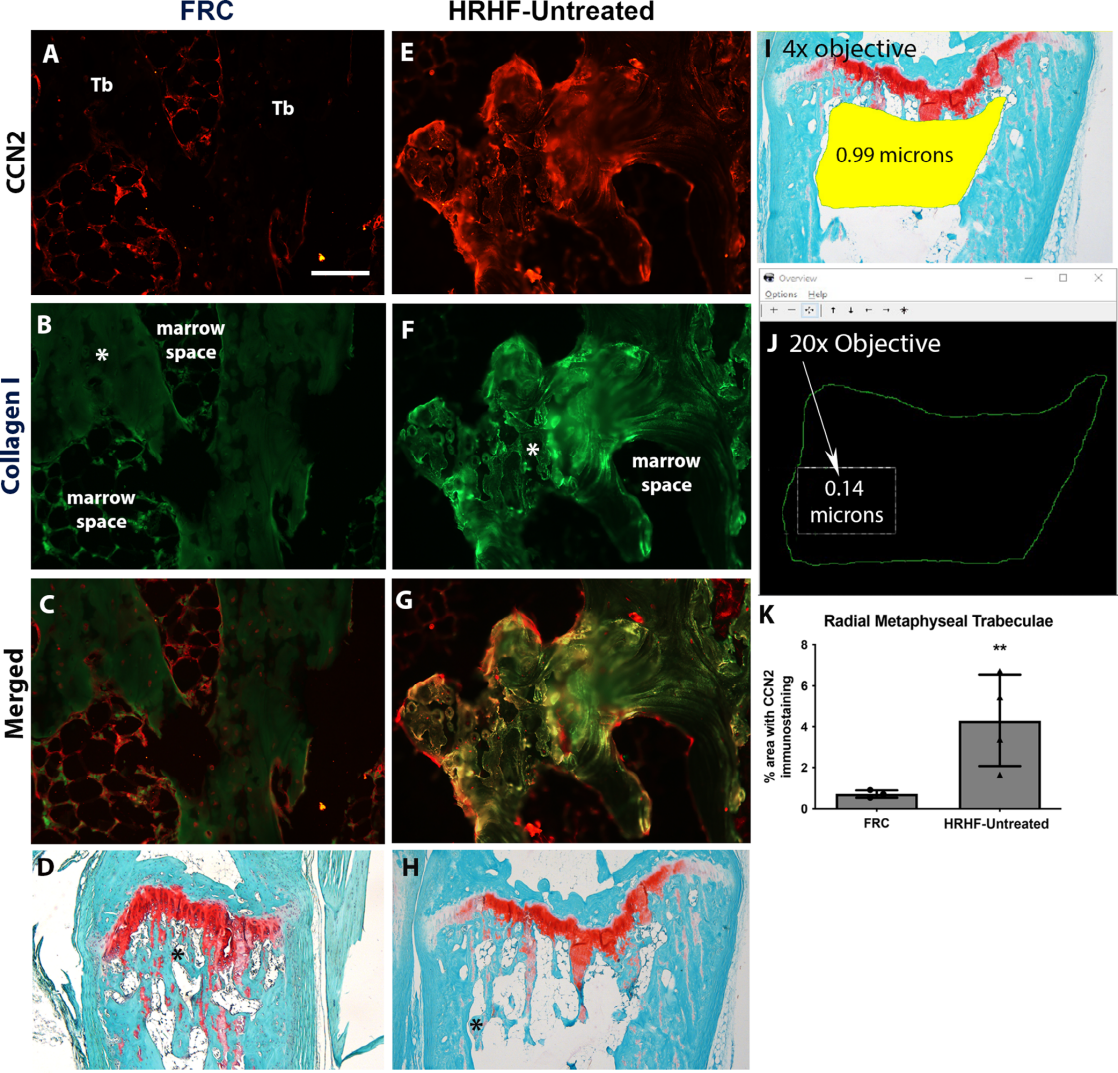
CCN2 immunostaining in distal radial trabecular bone. (*A*–*C*) Representative example of CCN2 (red) and collagen type I (green) immunostaining, and their merger, in a food‐restricted control (FRC) radial trabecular bone region. (*D*) Masson's trichrome‐stained section of FRC distal radius where an asterisk notes the specific region magnified in *A*–*C*. (*E*–*H*) Representative example of the same immunostaining and staining in a HRHF‐Untreated rat trabecular region where an asterisk in *H* notes the specific region magnified in *E*–*G*. (*A*) Scale bar = 50 μm (also applies to *B*, *C, E*–*G*). (*I*, *J*) Quantification method: Using a 4× objective, an irregular region of interest (ROI) in the distal radius trabecular bone region was circumscribed and landmarked for topographical mapping by an imaging system with a motorized stage. Next, using a 20× objective, a smaller rectangular region was used to systematically quantify the area of trabecular CCN2 immunoexpression within the boundaries of the larger irregular ROI. (*K*) Quantification of percent area with CCN2 immunostaining for distal metaphyseal trabecular region of the radius of FRC and HRHF‐Untreated animals. *p < 0.05 and **p < 0.01 compared with the FRC group.

Quantification of the percent area with CCN2 immunostaining was performed in the trabecular region of the distal radius using a thresholded videocount area quantification method.^[^ ^20^, ^37^ ^]^ For this, the microscope's exposure and gain choices were maintained at a constant level to ensure each acquired image was quantified similarly. A trabecular bone region located 150 μm below the distal radial growth plate and a few microns in from the surrounding cortical bone was circumscribed using the irregular region of interest (ROI) tool of the Bioquant imaging software at 40× magnification (using a 4× objective). The average area circumscribed was 1.4 μm^2^ (±0.65 SD) (Fig. 2*I*). A landmark is delineated (a step that informs the topographical mapping feature of the position of the motorized stage and that displays the chosen irregular ROI on the computer monitor; that is, the chosen topographical map; the larger irregular ROI shown in Fig. 2*I*, *J*). After landmarking, the objective was changed to 20× so as to sample at higher magnification (200× magnification), a rectangular ROI (0.14 μm^2^ in size, Fig. 2*J*) was used to systematically quantify the area of trabecular CCN2 immunoexpression within the boundaries of the larger irregular ROI. The Videocount Area Array option of the software was chosen (with video count area defined as the number of pixels in a field that meets a user‐defined color threshold of staining, multiplied by the area of a pixel at the selected magnification). Then, the area containing red pixels (CCN2 in this case) was quantified (with the output provided in microns^2^), followed by quantifying the total area of the field (all pixels in the field were chosen). Lastly, the area with red pixels was divided by the total area, and multiplied by 100, to give the percent area with CCN2 immunostaining in the chosen field. The trabecular bone within the boundaries of the larger irregular ROI was then quantified systematically for the percent area with CCN2 immunostaining, using the motorized stage. The person carrying out the immunohistochemical quantification was naive to group assignment.

### ELISAs

Serum samples from 6 to 7 FRC, 9 to 10 HRHF‐Untreated, 5 HRHF‐Rest/IgG, and 6 HRHF‐Rest/anti‐CCN2 rats were batch assayed for: (i) osteocalcin (a serum biomarker of osteoblastic activity,^[^ ^38^, ^39^, ^40^ ^]^ AC‐12F1, Rat‐MID Osteocalcin EIA, Immunodiagnostic Systems, Boldon, Tyne & Wear, UK); (ii) CTX‐1 (C‐telopeptide of type I collagen, a serum biomarker of bone resorption, AC‐06F1, CTX‐I EIA, Immunodiagnostic Systems, USA); (iii) estradiol levels (ES180S‐100, Calbiotech, El Cajon, CA, USA). ELISAs were conducted using manufacturers' protocols, in duplicate. The person carrying out the ELISA analyses was naive to group assignment.

### Statistical analyses

The sample size used for this study was derived from prior studies using this model,^[^ ^20^, ^21^, ^22^ ^]^ showing that at least *n* = 5/group was needed to detect bone morphometric changes using microCT or histomorphometry, assuming a power of 80% and a level of significance of 0.05 for BV/TV, osteoclast numbers, and osteoblast numbers.

GraphPad (La Jolla, CA, USA) PRISM v.9.5.1 was used for the statistical analyses. All data are expressed as mean ± SEM. The *p* values <0.05 were considered significant. Voluntary task parameters were compared using a mixed‐effects model (REML) and a repeated measures design (using the factors of group and task week). A Sidak post hoc test was then used, in which *p* values were adjusted for the multiple comparisons. Remaining data were tested for normality using the Shapiro–Wilk test. If the data were normally distributed, Brown–Forsythe ANOVAs were used, followed by Dunnett's 3 T post hoc tests. If data were not normally distributed, Kruskal–Wallis nonparametric ANOVAs were used, followed by Dunn's multiple comparisons post hoc tests. Adjusted *p* values are reported.

## Results

### Similar weight gains across weeks

Weight was carefully controlled in this longitudinal study (with no more than 5% loss in weight compared with age‐matched free‐access‐to‐food rats that were used for comparison purposes only). Rats were allowed to gain weight over the course of the experiment, as they were young adult rats at experimental onset. For this, all rats were weighed twice per week and were provided both regular rat chow daily and food reward pellets, or additional food as required. As shown in Supplemental Figure S1, each group gained weight similarly over time. Importantly here, both treatment groups gained weight similarly during the 6‐week rest period, ruling out weight differences as a contributor to any observed bone differences between the HRHF‐Rest/anti‐CCN2 versus HRHF‐Rest/IgG groups.

### Similar task performance between HRHF task groups

As shown in Figure 1, treatments did not begin until task cessation. Supplemental Figure S2 shows that rats in each of the three task groups performed the task similarly across the 18 weeks, a time point before euthanasia or the onset of the rest and drug treatments. Reach rate improved similarly across the weeks of task performance toward the target of 4 reaches/min (Supplemental Fig. S2 *A*), likely as a consequence of learning during the task period since this time period had no reach rate requirement (see Materials and Methods and our prior studies).^[^ ^7^, ^19^ ^]^ Only a few of the HRHF‐Rest/IgG rats reached the target reach rate by week 18 (Supplemental Fig. S2 *A*), although this did not reach significance compared with the other two task groups. All three task groups similarly met the target grasp‐force requirements, grasp duration, and reach impulse requirements in each task week (Supplemental Fig. S2 *B*–*D*). No statistically significant differences were found between groups in the mean number of reaches, grasp force, grasp duration, or reach impulse across the weeks.

### Long‐term HRHF performance increased CCN2 in the matrix of trabecular bone

Bones of 4 FRC and 4 HRHF‐Untreated rats were embedded in paraffin before microtome sectioning and then immunohistochemistry. We examined immunostaining of CCN2 in distal radius metaphyseal trabecular bone and radial diaphyseal cortical bone. In the FRC rats, CCN2 immunostaining was found in cells lining the trabeculae and in some cells in the bone marrow (Fig. 2*A*–*D*). In contrast, after long‐term HRHF task performance, CCN2 immunostaining was also found within the trabecular bone matrix and surrounding osteocytes (Fig. 2*E*–*H*). The immunostaining in FRC and HRHF‐Untreated rat bones was quantified (Fig. 2*I*–*K*) and showed increased CCN2 in the distal radial trabeculae and mid‐diaphyseal cortical bone of HRHF‐Untreated rats compared with FRC rats. We did not discriminate levels of CCN2 in the HRHF‐Rest/anti‐CCN2 group because the anti‐CCN2 antibody binding is thought to interfere with labeling of CCN2 present in tissues,^[^ ^41^ ^]^ as explained in detail in the discussion of one of our prior studies using this anti‐CCN2 agent.^[^ ^7^ ^]^

### Task‐induced declines in distal radial trabecular bone were improved by CCN2 blockade

**Fig. 3:**
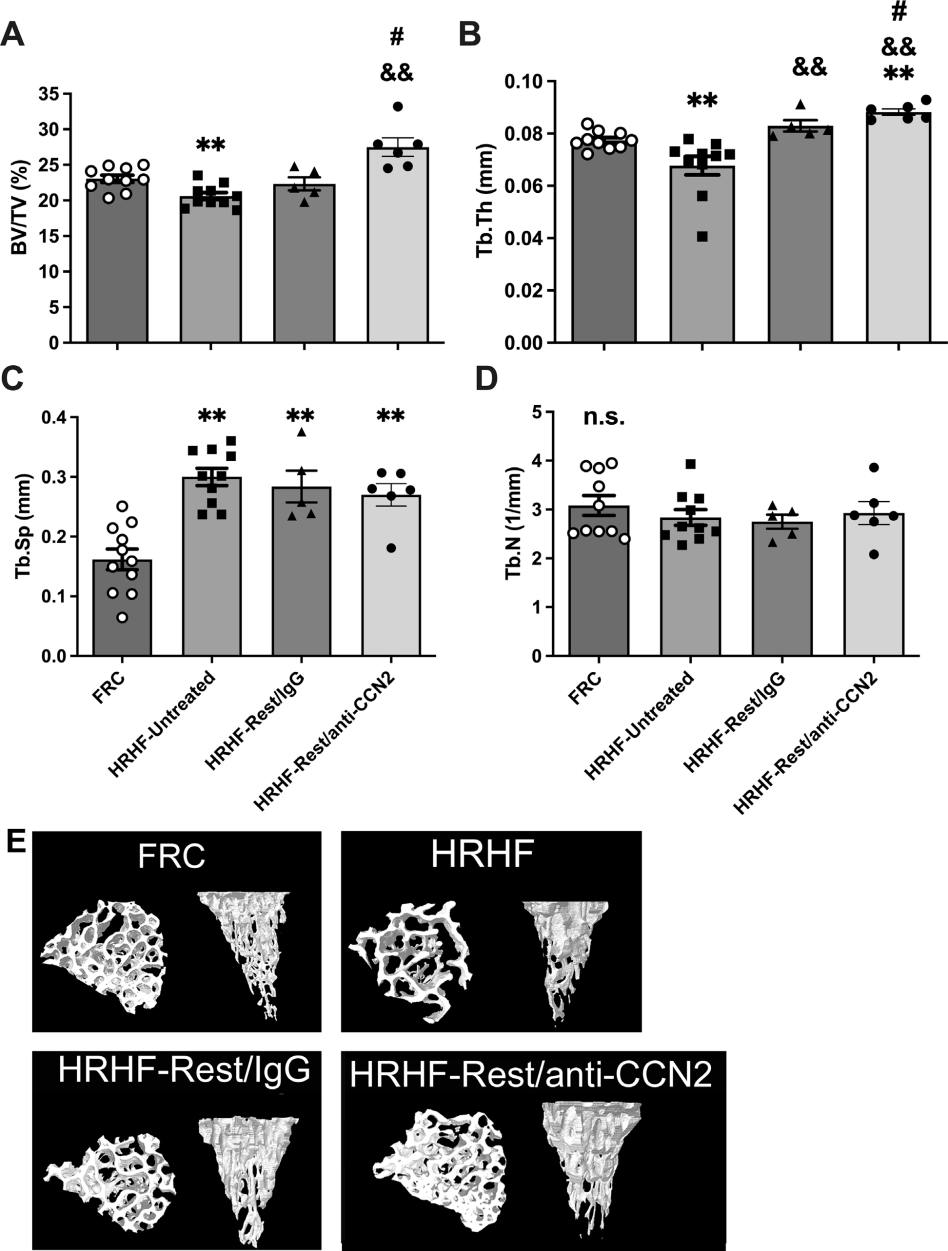
Micro‐computed tomography (microCT) of distal radial metaphyseal trabecular bone. (*A*) Trabecular bone volume fraction (BV/TV). (*B*) Trabecular thickness (Tb.Th). (*C*) Trabecular separation (Tb.Sp). (*D*) Trabecular number (Tb.N). Mean ± SEM shown. **p < 0.01 compared with FRC; ^&&^ *p* < 0.01 compared with HRHF‐Untreated; ^#^ *p* < 0.05 compared with HRHF‐Rest/IgG. n.s. = no significant difference between groups. (*E*) Representative transaxial and sagittal images of trabeculae in the distal radial metaphysis are shown for each group.

We have previously shown that prolonged performance of the HRHF task for up to 18 weeks leads to microarchitectural declines of trabeculae in the distal radial metaphysis, compared with age‐matched FRC rats.^[^ ^20^ ^]^ We extended that work here to examine the impact on this bone region of 6 weeks of rest after cessation of the 18‐week HRHF task, with simultaneous treatment of either a control IgG or the anti‐CCN2 antibody (as shown in the design, Fig. 1). MicroCT analysis showed a loss of trabecular bone volume fraction (BV/TV) in this bone region in HRHF‐Untreated rats compared with age‐matched FRC rats. Improvement in BV/TV was found in HRHF‐Rest/anti‐CCN2 rats compared with FRC and HRHF‐Untreated rats (Fig. 3*A*). Rest when combined with either treatment (IgG or anti‐CCN2) improved trabecular thickness (Tb.Th) compared with HRHF‐Untreated group (Fig. 3*B*), although this attribute was highest in the HRHF‐Rest/anti‐CCN2 group. Task‐induced increases in trabecular separation (Tb.Sp) were not rescued by either treatment (Fig. 3*C*). There were no significant differences in trabecular number (Tb.N) across groups (Fig. 3*D*). Representative 3D models of trabeculae from this region for each group are shown (Fig. 3*E*).

Thus, trabeculae in the distal radius of HRHF‐Untreated rats showed several catabolic changes. Six weeks of rest with either treatment improved Tb.Th (although more improvement was found in the HRHF‐Rest/anti‐CCN2 group compared with FRC). Importantly, HRHF‐Rest/anti‐CCN2 rats showed an increase in trabecular BV/TV and Tb.Th compared with HRHF‐Rest/IgG.

### Osteoblast numbers and osteoid width were most improved after CCN2 blockade; rest with either treatment reduced osteoclast numbers

**Fig. 4:**
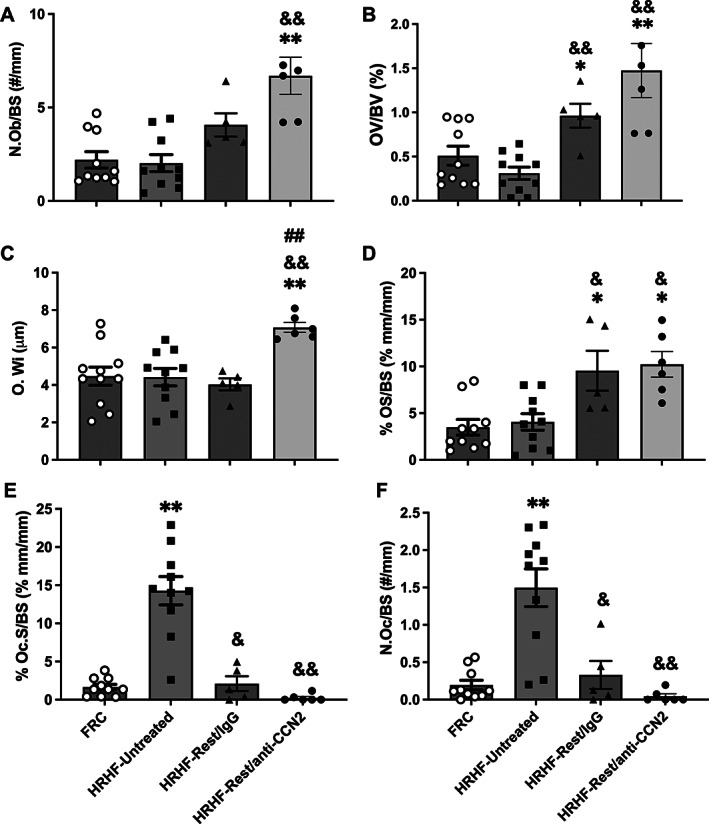
Static histomorphometry of osteoblast and osteoclast parameters in the distal radial metaphyseal trabecular bone. (*A*) Number of osteoblasts per bone surface (N.Ob/BS). (*B*) Osteoid volume (OV/BV). (*C*) Osteoid width (O.Wi). (*D*) Osteoid surface (OS/BS). (*E*) Osteoclast surface (Oc.S/BS). (*F*) Number of osteoclasts per bone surface (N.Oc/BS). Mean ± SEM shown. *p < 0.05 and **p < 0.01 compared with FRC group; ^&^ *p* < 0.05 and ^&&^ *p* < 0.01 compared with HRHF‐Untreated group; ^##^ *p* < 0.01 compared with HRHF‐Rest/IgG group.

To help elucidate mechanisms behind the microCT observations, static histomorphometry for osteoblast parameters was performed on trabeculae in this same bone region of the radius (Fig. 4*A*–*D*). Each osteoblastic attribute assayed using histomorphometry was similar in the HRHF‐Untreated rats and FRC groups (Fig. 4*A*–*D*), supporting a hypothesis of acclimation to task before the endpoint of 18 weeks. In contrast, numbers of osteoblasts per bone surface were highest in HRHF‐Rest/anti‐CCN2 radii compared with FRC and HRHF‐Untreated groups (Fig. 4*A*). Osteoid width was highest with CCN2 blockade (Fig. 4*C*) compared with the other groups. Rest, with either treatment, improved osteoid volume and osteoid surface compared with both the FRC and HRHF‐Untreated groups (Fig. 4*B*, *D*).

Next, static histomorphometry for osteoclast parameters was performed in the same distal metaphyseal trabecular region (Fig. 4*E*, *F*). Rest, with either treatment, ameliorated the task‐induced increases in osteoclast surface and number of multinucleated TRAP‐stained osteoclasts per bone surface.

Thus, examination of trabeculae in the distal radius at week 18 of the HRHF task (ie, HRHF‐Untreated rats) showed increased osteoclasts but not increased osteoblasts, the latter presumedly because of acclimation to the task by this time point. Osteoblast attributes were higher in the HRHF‐Rest/anti‐CCN2 group compared with FRCs, with osteoid width being the highest in this group compared with the other groups. In contrast, rest, with either treatment, lowered the task‐induced increases in osteoclast numbers.

### CCN2 blockade increased bone formation

**Fig. 5:**
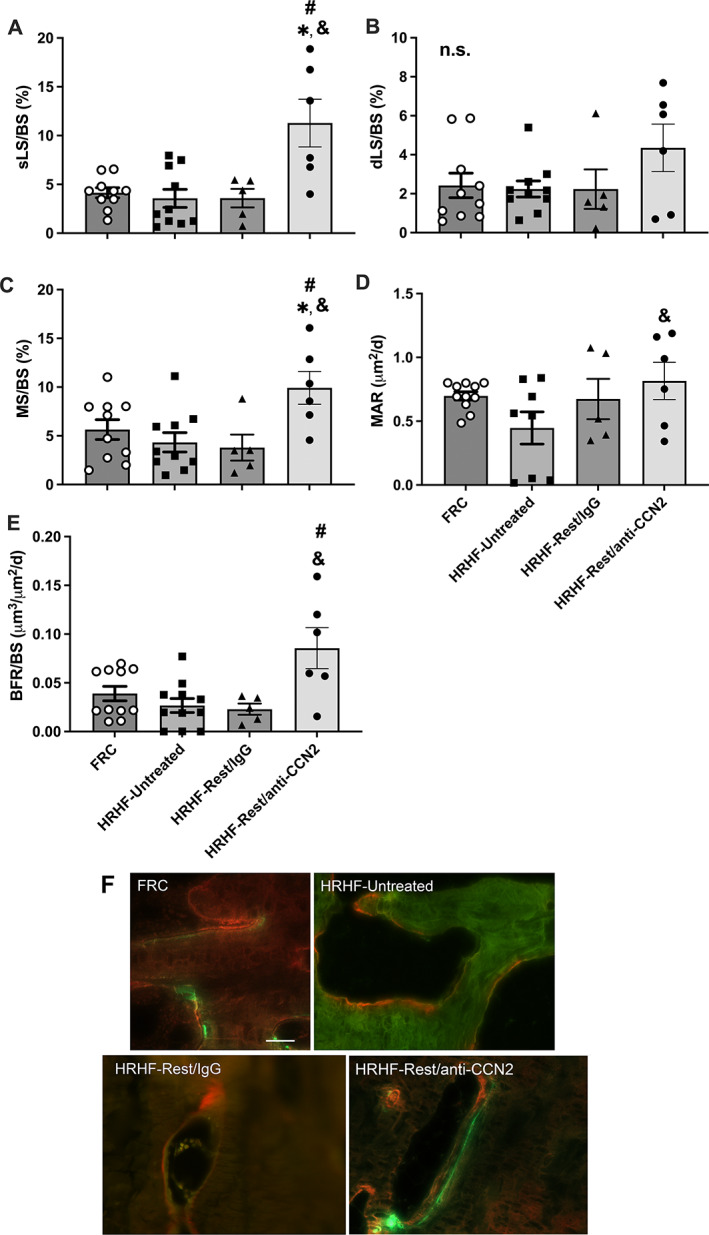
Dynamic histomorphometry of the distal radial metaphyseal trabecular bone. (*A*) Single‐labeled surfaces (sLS). (*B*) Double‐labeled surfaces (dLS). (*C*) Mineralizing surface (MS/BS). (*D*) Mineral apposition rate (MAR). (*E*) Bone formation rate (BFR/BS). Mean ± SEM shown. *p < 0.05 compared with FRC group; ^&^ *p* < 0.05 compared with HRHF‐Untreated group; ^#^ *p* < 0.05 compared with HRHF‐Rest/IgG group. n.s. = no significant difference between groups. (*F*) Representative images of calcein (green, injected 9 days before euthanasia) and alizarin red (injected 2 days before euthanasia) for each group, taken with a 40× objective. Scale bar (*F*, top left FRC panel) = 50 μm and is applicable to the other images in *F*.

Dynamic histomorphometry showed an increase in single‐labeled surfaces, mineralizing surfaces, and bone formation rate in the HRHF‐Rest/anti‐CCN2 group compared with the other groups (Fig. 5*A*, *C*, *E*), and increased mineral apposition rate (MAR) in the HRHF‐Rest/anti‐CCN2 group compared with the HRHF‐Untreated group (Fig. 5*D*). The double‐labeled surfaces did not differ between groups (Fig. 5*B*). Figure 5*F* shows representative fluorochrome images. Only trabeculae in the HRHF‐Rest/anti‐CCN2 rats show clearly distinct distances between the calcein line (green, injected 9 days before euthanasia) and the alizarin line (red, injected 2 days before euthanasia).

### Mid‐diaphyseal cortical bone was not affected by the task, rest, or CCN2 blockade

**Fig. 6:**
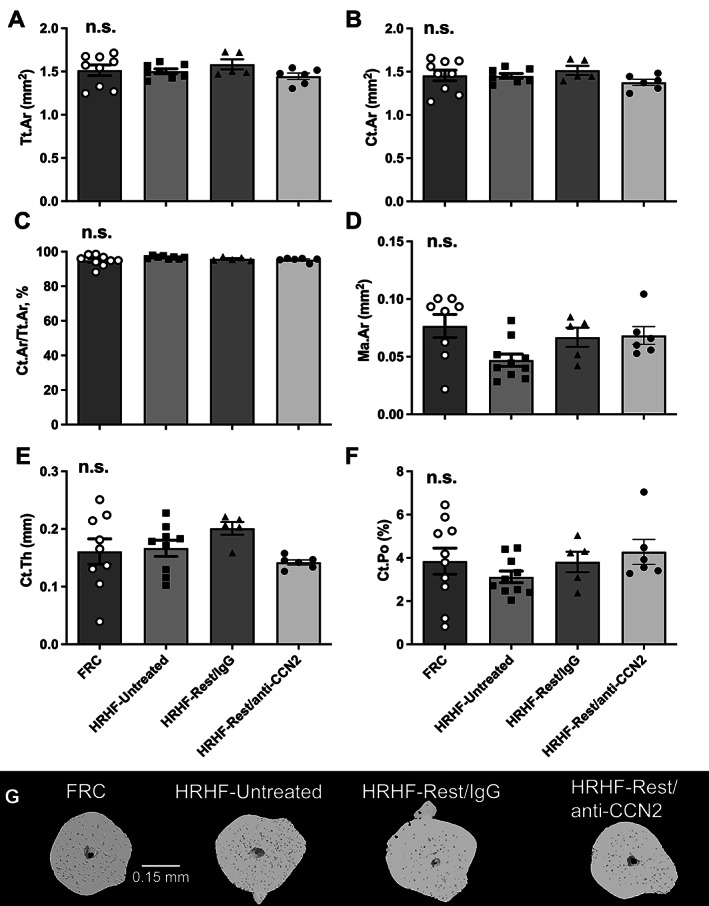
Micro‐computed tomography (microCT) of mid‐diaphyseal radial cortical bone. (*A*) Total cross‐sectional area inside the periosteal envelope (Tr.Ar). (*B*) Cortical bone area (Ct.Ar). (*C*) Cortical area fraction (Ct.Ar/Tt.Ar). (*D*) Marrow area (Ma.Ar). (*E*) Average cortical thickness (Ct.Th). (*F*) Cortical porosity (Ct.Po). Mean ± SEM shown. n.s. = no significant difference between groups. (*G*) Representative 3D models of the mid‐diaphyseal cortical bone in each group, shown as transaxial images.

We also examined the microarchitecture of mid‐diaphyseal cortical bone of the radius using microCT (Fig. 6). No significant differences were observed between the groups for any attribute examined. Figure 6*G* shows representative 3D models of the mid‐diaphyseal cortical bone in each group.

### Serum bone biomarker outcomes show blocking CCN2 signaling increases osteoblast activity yet decreases osteoclast activity

**Fig. 7:**
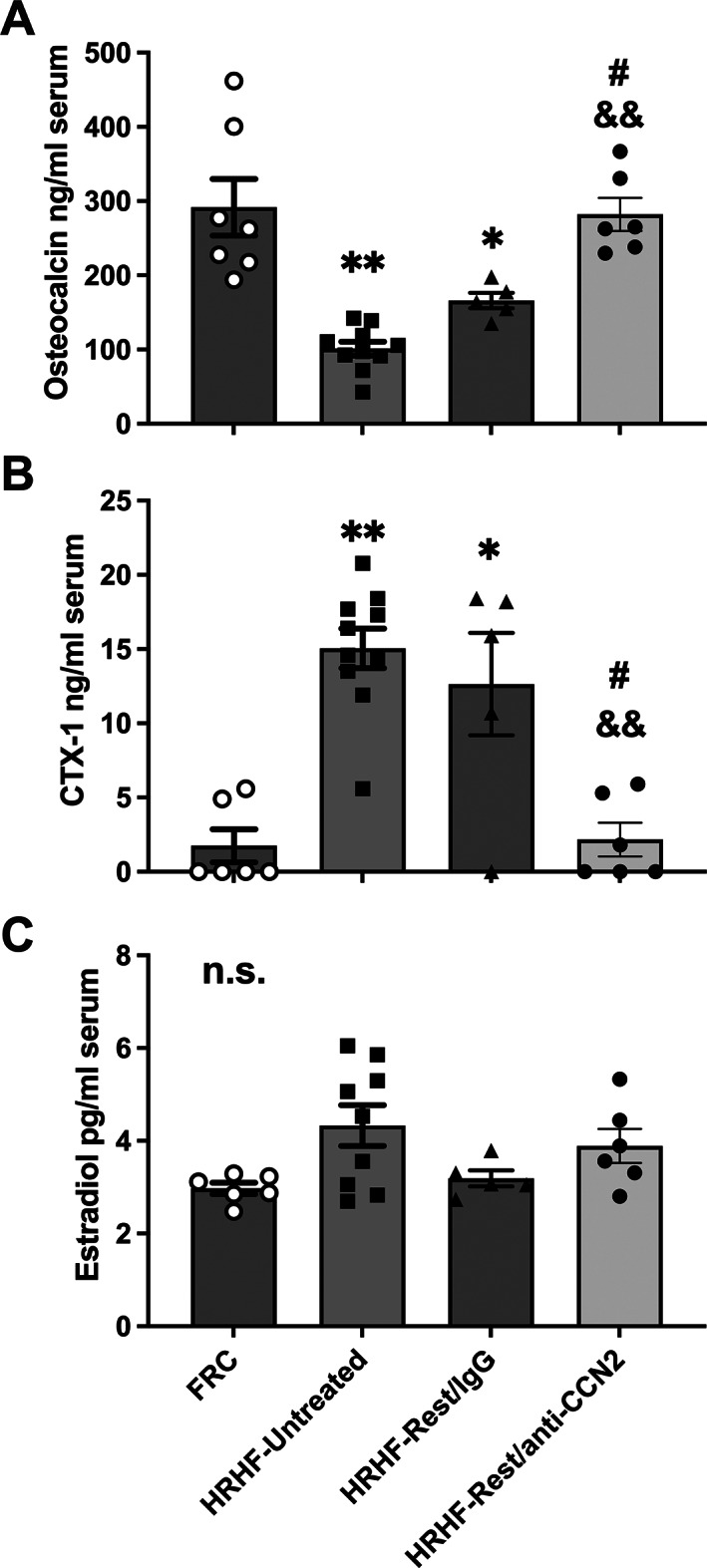
Serum levels of bone turnover biomarkers and estradiol levels. (*A*) Osteocalcin. (*B*) C‐telopeptide of type I collagen (CTX‐1). (*C*) Estradiol. Mean ± SEM shown. **p < 0.01 compared with FRC group; ^&&^ *p* < 0.01 compared with HRHF‐Untreated group; ^#^ *p* < 0.05 compared with HRHF‐Rest/IgG group. n.s. = no significant difference between groups.

Task‐induced declines in serum osteocalcin levels (a serum biomarker of osteoblastic activity) was elevated (improved) in the HRHF‐Rest/anti‐CCN2 rats compared with the HRHF‐Untreated and HRHF‐Rest/IgG groups (Fig. 7*A*). Task‐induced increases in serum CTX‐1 levels (a serum biomarker of osteoclast‐driven bone resorption) were reduced (ameliorated) in the HRHF‐Rest/anti‐CCN2 rats but not in the HRHF‐Rest/IgG rats (Fig. 7*B*). Serum estradiol was also assayed for any effects of task or treatment and showed no statistically significant differences across groups (Fig. 7*C*).

## Discussion

This is the first study examining the effects of CCN2 blockade on reversing established bone loss. We used our model of upper‐extremity overuse injuries to study the effect of long‐term (18‐week) HRHF task performance on underlying bone microarchitecture and function and whether these could be altered by blocking CCN2 after cessation of the task, and, if so, through which mechanism(s). We have previously shown that early use of an anti‐CCN2 drug (FG‐3019) prevents bone loss induced by short‐term (3‐week) HRHF task performance.^[^ ^7^ ^]^ This occurred primarily through inhibition of osteoclastogenesis and activity. In this current study, CCN2 deposition was dramatically increased in the matrix of trabeculae in the distal radii after 18 weeks of HRHF task performance. HRHF‐Untreated rats showed several catabolic changes in trabeculae in the distal radius, including reduced trabecular bone volume fraction and thickness, as well as increased trabecular separation, osteoclast numbers, and serum CTX‐1. However, there were no changes in osteoblast numbers in the HRHF‐Untreated rats from FRC levels, presumably because of acclimation to the task after so many weeks of performance. Only the HRHF‐Rest/anti‐CCN2 group showed an increase in trabecular BV/TV, likely attributable to the improvement in Tb.Th. Most of the osteoblast attributes were higher in the HRHF‐Rest/anti‐CCN2 group compared with FRCs, with osteoid width being highest in HRHF‐Rest/anti‐CCN2 group, as was bone formation rate. Rest with either treatment lowered the task‐induced increases in osteoclast numbers.

Work from our lab has previously showed that performance of an intense HRHF task results in trabecular bone loss.^[^ ^7^, ^19^, ^20^, ^21^, ^22^ ^]^ When the distal radius bone is exposed to cyclical loading with high‐magnitude force as a consequence of this lever‐pulling task, it is apparently unable to sustain bone production commensurate with task‐induced loss.^[^ ^20^, ^42^ ^]^ This is consistent with the Fatigue‐Failure theory for musculoskeletal disorder injuries.^[^ ^19^, ^42^, ^43^ ^]^ Clinically, this is particularly salient in the diagnosis and treatment of overuse injuries. Most often, patients present after developing symptoms from sustained musculoskeletal disorders (eg, tendonitis, carpal tunnel syndrome, and stress fractures).^[^ ^42^ ^]^ The availability of adjunct therapies during recovery may allow faster return to work and mitigate further disability.

Similar to a past study examining bone responses to short‐term loading,^[^ ^7^ ^]^ we found that long‐term exposed HRHF rats showed a significant increase in CCN2 expression in the distal radial metaphysis, although the presence of CCN2 was more apparent in the bone matrix by 18 weeks. We did not further discriminate levels of CCN2 in the pharmacologic treatment groups because the anti‐CCN2 antibody binding is thought to interfere with labeling of CCN2 present in tissues,^[^ ^41^ ^]^ as explained in detail in the discussion of one of our prior studies using this anti‐CCN2 agent.^[^ ^7^ ^]^ Yet, based on these immunohistochemical results in bone, we assessed the effects of CCN2 blockade in our long‐term HRHF model on bone microarchitecture and histomorphometry.

The anti‐CCN2 drug, FG‐3019, branded as Pamrevlumab, is a fully human monoclonal antibody against CCN2 (FibroGen, Inc.). Its mechanism specifically involves targeting the von Willebrand factor C domain of the CCN2 protein.^[^ ^41^ ^]^ Pamrevlumab has been granted US Food and Drug Administration (FDA) Fast Track and Orphan Drug Designation for use in locally advanced pancreatic carcinoma, Duchenne muscular dystrophy, and idiopathic pulmonary fibrosis and has entered into phase 3 clinical trials for these indications.^[^ ^23^ ^]^ Results of phase 2 trials have thus far shown potential for decreasing tissue fibrosis with functional improvements.^[^ ^24^, ^25^ ^]^ No clinical trials to date have noted its effect on bone. Human safety trials exhibit low rates of adverse events and support that anti‐CCN2 may be safe and well tolerated in the target patient population. There are other several clinically approved anabolic treatments, specifically the parathyroid hormone analogs teriparatide (Forteo) and abaloparatide (Tymlos), as well as the anti‐sclerostin antibody romosozumab (Evenity). All three are approved for the treatment of postmenopausal osteoporosis in women at high risk for fracture.^[^ ^44^, ^45^, ^46^ ^]^ Our series of studies studying the effect of CCN2 inhibition in neuromusculoskeletal tissues was performed using young, female Sprague–Dawley rats. To apply the aforementioned anabolic therapies to our overuse injury model in keeping with FDA‐approved indications, one would need to utilize a validated postmenopausal model (eg, an ovariectomized rat that has some key differences from human female menopause, including a sudden loss of estradiol,^[^ ^47^ ^]^ which is beyond the scope of this study). To our knowledge, there are no current data utilizing these pharmacotherapies in the treatment of overuse‐induced bone loss.

We have previously used the anti‐CCN2 drug in our overuse model during short‐term (3‐week) HRHF performance. We found a decrease in distal radius bone loss through a reduction in osteoclastogenesis and bone resorption.^[^ ^7^ ^]^ This finding was consistent with prior work showing that CCN2 enhances RANKL and DC‐STAMP pathways in osteoclast formation and maturation.^[^ ^16^, ^17^, ^48^ ^]^ Here for the first time, we show that anti‐CCN2 treatment can reduce established bone loss through primarily enhanced osteoblast function (although perhaps also some decreases in osteoclast function, based on the serum CTX‐1 results). At the cellular level, anti‐CCN2 therapy after completion of HRHF task resulted in increased osteoid width compared with the HRHF‐Rest/IgG group, and increased osteoblast numbers, osteoid volume, and osteoid width compared with the HRHF‐Untreated and Control groups. It has been shown that in osteoblasts, CCN2 expression increases during the early stages of osteoblast proliferation and differentiation and then later decreases during terminal differentiation. Studies by our group, Safadi and colleagues^[^ ^,(6)^ ^]^ and others have showed that the administration of recombinant CCN2 to osteoblasts or osteoblastic cell lines (eg, MC3T3‐E1 cells) promotes proliferation and upregulation of bone formation markers such as type I collagen, alkaline phosphatase, osteopontin, and osteocalcin. However, constitutive overexpression has the opposite effect, inhibiting BMP‐induced osteoblast differentiation.^[^ ^2^, ^13^ ^]^ This has further been shown in a transgenic mouse model of CCN2 overexpression under the osteocalcin promoter (affecting mature osteoblasts and osteocytes), which caused osteopenia due to impaired osteoblast activity, specifically decreased mineral apposition and bone formation rates.^[^ ^14^ ^]^ We have shown in this article and a prior publication from our lab that CCN2 expression is significantly increased in bone, including in and around osteocytes, after HRHF task performance.^[^ ^7^ ^]^ Therefore, we believe that our findings do correlate with prior studies in which overexpression of CCN2 in mature bone cells results in osteopenia and that by blocking this with anti‐CCN2 treatment, we are reducing the inhibitory effects of CCN2 on osteoblast function and bone formation, observed in HRHF‐Untreated rats as reduced bone mass. Blocking CCN2 during the rest period reduced the inhibitory effects of CCN2 on osteoblast function and bone formation, observed as a reduction of the loss in bone found in HRHF‐Untreated and HRHF‐Rest/IgG groups. This most likely occurs at the level of the bone milieu and not systemically, as we have previously shown that serum CCN2 levels remain elevated in both rest groups.^[^ ^27^ ^]^ Similar changes were not found in cortical bone after rest. This is likely because of lower turnover in this region of these mature rats and corresponds with prior studies by our group and others.^[^ ^20^, ^49^, ^50^ ^]^

The contributions of stimuli generated by muscle on bone are still being examined in the literature.^[^ ^51^, ^52^, ^53^ ^]^ We saw no differences in task performance measures between rats in the three HRHF groups during the first 18 weeks of the experiments, as expected since treatments did not start after completion of the 18‐week task. Therefore, differences in task performance before treatment onset can be ruled out as a potential contributor. Yet, changes in neuromuscular forelimb tissues may be potential contributors to the observed bone changes. We have shown in these same HRHF‐Rest/anti‐CCN2 rats that task‐induced increases in muscular, dermal, and neural collagen deposition and fibrosis were reversed by blocking CCN2 during the rest period.^[^ ^27^, ^34^ ^]^ These rats also had improved functional declines (including improved grip strength and median nerve conduction velocity, and reduced cold temperature sensitivity).^[^ ^27^, ^34^ ^]^ The improved grip strength correlated negatively and moderately with muscle levels of TGF‐beta1, CCN2, and bFGF,^[^ ^27^ ^]^ proteins involved in bone signaling and metabolism.^[^ ^2^, ^17^, ^54^ ^]^ Thus, skeletal muscle‐bone cross‐talk may be contributing to some of the improved bone parameters. We have also shown rescue of neuronal stress and sensitization in the form of reduced activating transcription factor 3 (ATF‐3) in spinal cord motor neurons (a sign of neuronal stress) and decreased production of Substance P by dorsal root ganglia neurons (a sign of sensory neuron sensitization) in these same HRHF‐Rest/anti‐CCN2 rats. Therefore, there may also be a nerve function contribution to the observed bone remodeling.^[^ ^55^ ^]^ That said, the rest period, with or without CCN2 blockade, resulted in a decreased osteoclastic response. This would be consistent with findings from another study in which we examined the effects of 6 weeks of rest, with or without a manual therapy treatment, after a 12‐week task period.^[^ ^31^ ^]^ We found that rest (alone or combined with manual therapy) effectively rescued the osteoclastic response but did not improve osteoblastic numbers or activity or already established task‐induced loss in trabecular bone volume fraction.^[^ ^31^ ^]^ Only the anti‐CCN2 treatment during rest improved the task‐induced trabecular bone loss.

Limitations of our study include that we examined bones from only female rats. Adult female rats were used for several reasons: (i) human females have a higher incidence of work‐related overuse disorders^[^ ^56^, ^57^, ^58^ ^]^ (ii) our injury model is currently tailored to the pulling strength of female rats; and (iii) tissues evaluated in this study were collected from female rats from which remaining forelimb tissues were examined as part of another study. Inclusion of males would have reduced data quality and made the interpretation of findings difficult. To somewhat counter this potential limitation, we examined whether serum estradiol levels were altered by either task or treatment. As shown in Figure 7, there was no significant change in serum estradiol levels between the groups. This corresponds to our findings in prior studies examining the effects of the HRHF task for 3 to 18 weeks.^[^ ^7^, ^27^ ^]^ We also did not study the effects of anti‐CCN2 on bones of FRC rats and so cannot determine if there were bone effects on animals that did not perform the HRHF task. Prior studies have shown that CCN2 as a matricellular protein has skeletal site‐specific and context‐dependent actions in bone.^[^ ^12^, ^59^ ^]^ It is therefore likely that findings in FRC + anti‐CCN2 rats may show effects that significantly differ from those after HRHF task performance. As prior clinical studies have not investigated the effects of anti‐CCN2 on bone, it is unknown whether a difference occurs in human control groups. Additionally, we chose to assess bone structure in the radius as it is the primary load‐bearing forelimb bone in force transmission. We have previously studied changes in other bones involved in upper‐extremity force transmission (ulna and humerus) and found the greatest changes in the radius,^[^ ^21^, ^60^, ^61^ ^]^ although the ulna and humerus also undergo task‐related changes.^[^ ^21^, ^60^, ^61^ ^]^ We have also observed presumed systemic changes in hindlimb bones as a consequence of task‐induced increases in circulating inflammatory cytokines.^[^ ^60^ ^]^ Changes in other forelimb bones and a potential systemic effect could explain the inconsistency between osteoclast histomorphometric measurements (Fig. 4*E*, *F*) and circulating CTX‐1 levels (Fig. 7*B*). That said, histomorphometric data showed increased number of osteoblasts and decreased numbers of osteoclasts, respectively, which we believe aligns with our finding of increased osteocalcin levels in support of bone remodeling with growth and decreased serum CTX‐1 levels showing a decrease in bone resorption in the anti‐CCN2‐treated animals.

We did not assay for PINP in this study, a marker of collagen type I synthesis by many cell types, including osteoblasts, fibroblasts, and other cells that make collagen type I. PINP has been shown to be an excellent serum biomarker of fibrosis (ie, collagen type I synthesis) in patients with dilated cardiomyopathy and different grades of diastolic dysfunction of the left ventricle,^[^ ^62^ ^]^ idiopathic pulmonary fibrosis,^[^ ^63^ ^]^ liver matrix remodeling with fibrosis,^[^ ^64^ ^]^ and muscle fibrosis associated with muscular dystrophies and other myopathies.^[^ ^65^ ^]^ Because our model induces muscle, tendon, and nerve fibrosis, we see serum increases of PINP as part of the muscle and other soft tissue collagen production and fibrosis,^[^ ^27^ ^]^ a finding that confounds the interpretation of PINP as a marker of bone formation in this model.

In conclusion, treatment of HRHF rats with 6 weeks of rest combined with anti‐CCN2 reduced the catabolic bone effects of task performance. Although rest with either treatment decreased resorption, the anti‐CCN2 treatment enhanced bone formation more than the IgG treatment.

## Author Contributions

**Alex G Lambi:** Conceptualization; formal analysis; visualization; writing – original draft; writing – review and editing. **Michele Y Harris:** Investigation; methodology; validation; writing – review and editing. **Mamta Amin:** Formal analysis; investigation; methodology; validation; writing – review and editing. **Patrice G Joiner:** Formal analysis; investigation; methodology; validation; writing – review and editing. **Brendan A Hilliard:** Validation; visualization; writing – review and editing. **Soroush Assari:** Data curation; software; writing – review and editing. **Steven N Popoff:** Conceptualization; visualization; writing – original draft; writing – review and editing. **Mary F Barbe:** Conceptualization; methodology; validation; formal analysis; investigation; writing – original draft; writing – review and editing; visualization; supervision; project administration; funding acquisition.

## Funding Statement

This work was supported by the National Institute of Arthritis and Musculoskeletal and Skin Diseases (NIAMS) of the National Institutes of Health under grant AR056019 to MFB. The content is solely the responsibility of the authors and does not necessarily represent the official views of the National Institutes of Health.

## Conflict of Interest

The authors have no financial conflicts of interest or disclaimers to declare.

### Peer Review

The peer review history for this article is available at https://www.webofscience.com/api/gateway/wos/peer-review/10.1002/jbm4.10783.

## Supporting information


**Supplemental Fig. S1.** Body weight in centinewtons (cN) across the weeks of shaping and HRHF task performance for each group, as well as during the 6‐week rest period for the HRHF‐Rest/IgG and HRHF‐Rest/anti‐CCN2 groups.

**Supplemental Fig. S2.** Task performance across the weeks of HRHF task performance for each group before the onset of rest and drug treatments. (*A*) Number of reaches/minute. (*B*) Mean grasp force (cN). (*C*) Mean grasp duration (msec). (*D*) Mean reach impulse. Expected targets or ranges are indicated for each measure with dotted lines. n.s. = not significant.
