# Supplementary figures and images for: Blocking CCN2 Reduces Established Bone Loss Induced by Prolonged Intense Loading by Increasing Osteoblast Activity in Rats
Source: JBMR Plus. 2023 Jun 16;7(9):e10783. doi: 10.1002/jbm4.10783 (PMC10494513; doi:10.1002/jbm4.10783)

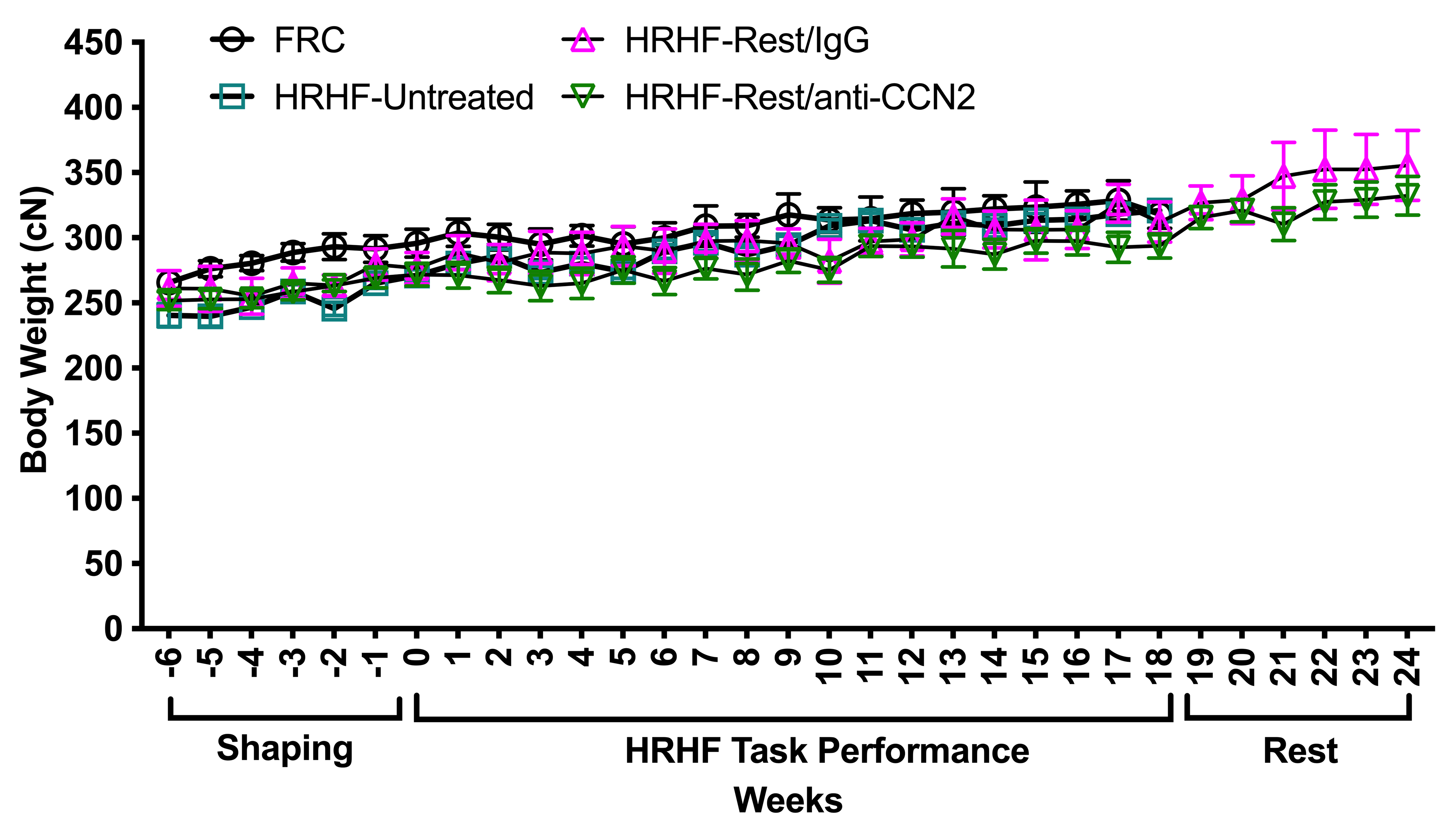

Supplement: Supplementary file 1 — Supplemental Fig. S1. Body weight in centinewtons (cN) across the weeks of shaping and HRHF task performance for each group, as well as during the 6‐week rest period for the HRHF‐Rest/IgG and HRHF‐Rest/anti‐CCN2 groups. [file JBM4-7-e10783-s001.tif]

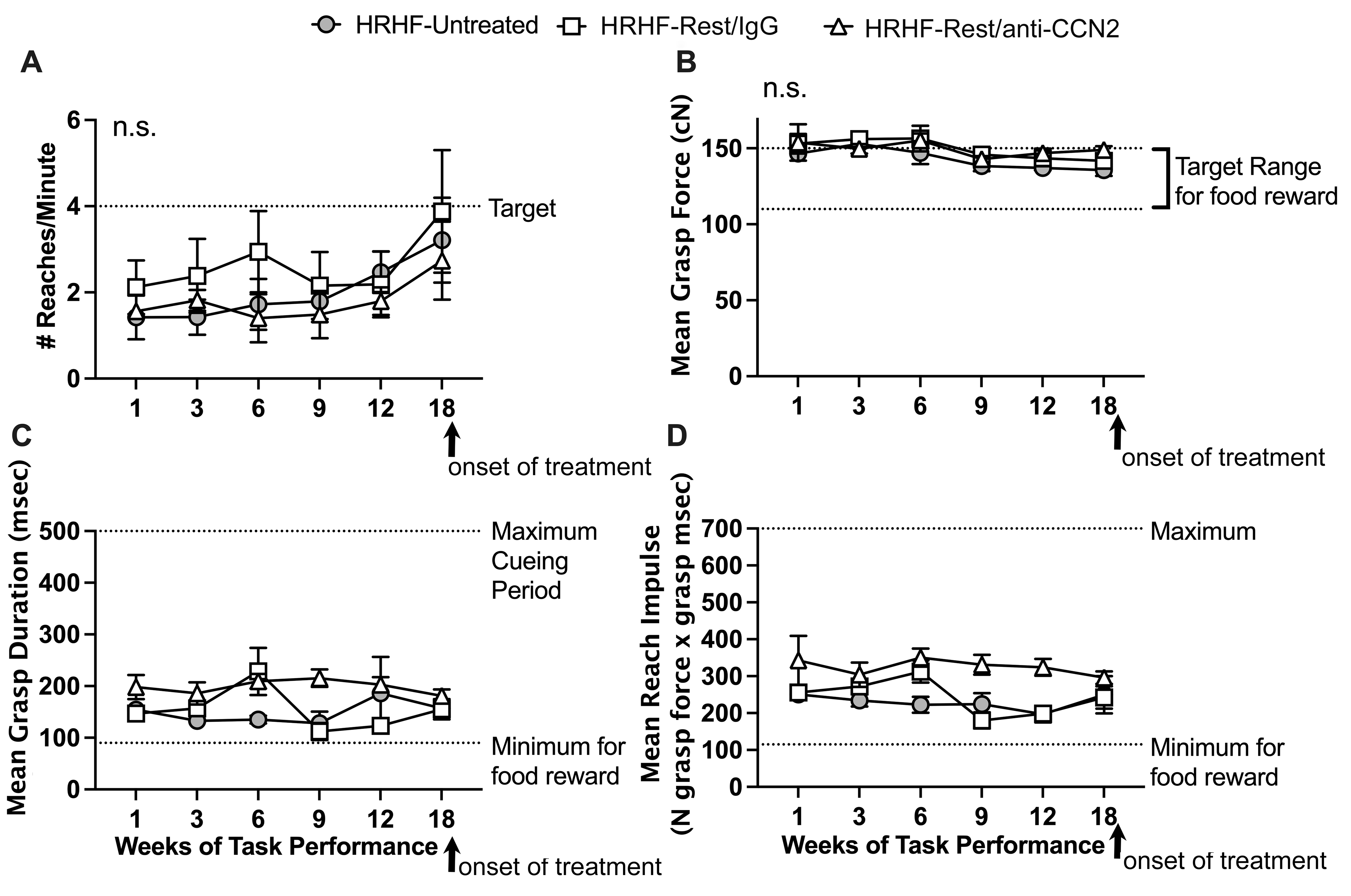

Supplement: Supplementary file 2 — Supplemental Fig. S2. Task performance across the weeks of HRHF task performance for each group before the onset of rest and drug treatments. (A) Number of reaches/minute. (B) Mean grasp force (cN). (C) Mean grasp duration (msec). (D) Mean reach impulse. Expected targets or ranges are indicated for each measure with dotted lines. n.s. = not significant. [file JBM4-7-e10783-s002.tif]
